# ToF Intensity-Guided Cross-Modal Region of Interest Generation for Metal Surface Defect Detection

**DOI:** 10.3390/s26196243

**Published:** 2026-10-01

**Authors:** Gyusang Jang, Sungan Yoon, Yosup Lee, Jeongho Cho

**Affiliations:** 1 Department of Electrical Engineering, Soonchunhyang University, Asan 31538, Republic of Korea; 20267026@sch.ac.kr (G.J.); ysa258@sch.ac.kr (S.Y.); 2 CMOS Media Tech Co., Ltd., 397 Seokcheon-ro, Bucheon 14449, Republic of Korea; lucas@cmosmedia.com

**Keywords:** Time-of-Flight sensing, RGB imaging, homography, automatic surface inspection, metal surface defect detection, object-adaptive region of interest

## Abstract

High-speed mass-production environments require reliable surface inspection because metal surface defects directly affect product reliability and process stability. Recent automatic surface inspection systems have increasingly adopted RGB image-based deep learning detectors to identify defects such as scratches, dents, and contamination. However, in practical inspection settings, the position and pose of small metal components can vary from frame to frame, and the reflective nature of metallic surfaces can make background texture visually similar to defect patterns. This study presents a cross-modal Region of Interest (ROI) generation framework that combines Time-of-Flight (ToF) intensity images with RGB images to define a stable component-level ROI before defect detection. The proposed method exploits signal attenuation and dropout patterns in ToF intensity images, caused by specular reflection on metallic surfaces, as structural cues for object localization. Foreground support regions extracted from the ToF intensity image are separated into object candidates using connected-component analysis and then projected onto the RGB coordinate system through a pre-calibrated homography. An object-adaptive ROI is generated from the projected region and provided to a YOLO-based defect detector. Experimental results show that the proposed method improves mAP@50 from 0.585 to 0.796 compared with the baseline YOLO26 model, demonstrating that ToF-guided object-adaptive ROI generation effectively reduces background interference and improves localization stability in metal surface defect detection.

## 1. Introduction

In high-speed mass-production environments, the surface quality of metal components is a critical factor that determines functional reliability and process stability [1,2]. Surface defects such as scratches, dents, and oil contamination can lead to assembly failure, functional degradation, and poor appearance quality regardless of defect size. Therefore, production lines require automatic surface inspection systems (ASIS) based on full inspection to detect such defects rapidly and consistently [3,4,5]. In particular, when small components of only a few centimeters move through the inspection area, not only the performance of the defect detector but also the ability to separate and align each component as a stable analysis unit before detection becomes an important factor in overall inspection reliability.

Previous ASIS studies have largely focused on improving defect detection accuracy from RGB images containing surface defects. Early approaches relied on rule-based image processing using low-level visual cues such as intensity contrast, edges, and texture. However, such methods show limited generalization in real industrial images where defects vary in shape and have low contrast. To overcome these limitations, deep learning-based methods using object detection and segmentation models have been widely investigated [6,7,8]. Among them, You Only Look Once (YOLO) detectors are commonly used for metal surface defect detection because they provide a favorable balance between speed and accuracy [9,10,11]. Nevertheless, these approaches generally assume that the input image for defect detection is already aligned around a single component or that a stable Region of Interest (ROI) has been obtained. In other words, many studies focus on improving detector architectures, whereas component position variation and background inclusion before defect detection in real production environments have not been sufficiently addressed.

Although the defects themselves are static, production-line inspection is performed on successive components whose positions and poses vary from frame to frame. The component-level ROI must therefore be localized for each image before defect detection. On reflective metal surfaces, this step is further complicated by specular reflection, overexposure, and nonuniform surface texture [12,13,14]. If a component-level ROI is unstable, the detector receives not only the component surface but also irrelevant regions such as the background, rails, rubber mats, and lighting reflections. In this case, background texture or reflection patterns can act as visual features similar to defects, increasing false positives and localization errors. Oil contamination is especially challenging because it has no fixed shape or clear boundary and often appears as a weak, local signal. Some studies have attempted to improve defect representation using segmentation or feature representation; however, these methods generally assume that the input region for defect detection has already been stably acquired [15,16]. Therefore, stable component-level ROI generation before defect detection remains an important technical bottleneck in ASIS.

To address this problem, this study uses the intensity image from a Time-of-Flight (ToF) sensor to stabilize the ROI before defect detection, rather than relying solely on RGB images. On metallic surfaces, specular reflection often makes ToF depth values unstable and repeatedly generates signal attenuation or dropout patterns in the intensity image. Although such signals are commonly treated as sensor noise or as artifacts requiring correction, this study focuses on their potential as structural cues that reflect the spatial support and location of metal components. Here, the ToF channel is used specifically for component localization, while defect appearance is evaluated in the RGB image. In other words, ToF determines where the component-centered ROI is placed rather than serving as a defect feature. This is useful on reflective metal surfaces, where RGB appearance alone can be strongly affected by specular reflection and background texture.

This paper proposes a cross-modal object-adaptive ROI generation framework that combines ToF intensity and RGB images. The proposed method first extracts foreground support regions from the ToF intensity image based on signal attenuation and dropout patterns, and then separates valid object candidates using connected-component analysis. The extracted ToF foreground pixels are directly projected onto the RGB coordinate system using a fixed homography obtained through prior calibration. The spatial extent of the projected foreground support is used to generate an object-adaptive bounding box, which reconstructs the defect detection input as a component-centered ROI even when component position changes occur. Because this process does not require complex multimodal feature fusion or frame-by-frame feature matching, it is computationally lightweight and can be easily integrated into existing RGB-based inspection systems as a preprocessing module.

The central idea of the proposed approach is not to make the detector architecture more complex, but to reconfigure the spatial distribution of the detector input around the target component. When a full RGB image is used directly, the defect region occupies only a very small portion of the high-resolution image, and surrounding background texture and reflection patterns may dilute the defect features. By contrast, the proposed object-adaptive ROI suppresses background inclusion and increases the relative proportion of the component surface where defects may exist. As a result, the detector can focus more on local intensity variations and texture differences associated with defects, improving detection stability for low-contrast contamination and irregular damage defects.

The main contributions of this paper are summarized as follows.

1. First, ToF intensity dropout patterns occurring on metallic surfaces are reinterpreted as structural cues for extracting component foreground support rather than as simple sensor noise. This enables object separation and position estimation on reflective metal surfaces where depth values can be unstable.
2. Second, a lightweight ToF-RGB geometric ROI generation pipeline is implemented. The pipeline directly projects ToF foreground pixels onto the RGB coordinate system using a pre-calibrated fixed homography and generates object-adaptive bounding boxes from the spatial extent of the projected foreground support. This process does not require frame-by-frame feature matching or complex multimodal feature fusion.
3. Third, the proposed ROI generation module is formulated as a detector-agnostic preprocessing stage that reconfigures the input image around the component without modifying the architecture of existing RGB-based detectors. Quantitative experiments demonstrate that this strategy reduces background texture and reflection interference and improves the detection performance for low-contrast contamination and irregular damage defects.

## 2. Related Work

This section reviews previous studies on ASIS from the perspectives of problem formulation and sensor utilization. First, the development of RGB image-based metal surface defect detection methods is summarized. Then, approaches using additional sensors such as depth, photometric stereo, and ToF are discussed. Based on this review, we show that previous studies have mainly focused on defect representation or detector performance, whereas the component-level ROI stabilization required before defect detection has been relatively underexplored.

### 2.1. Metal Surface Defect Detection Techniques

Surface defect inspection and dynamic-system fault diagnosis both seek to identify abnormal conditions from measured data, but they generally use different forms of information. Fault diagnosis typically analyzes temporal signals related to system behavior, whereas surface inspection focuses on spatial image patterns on manufactured parts. Recent reviews have summarized the growing use of learning-based and generative approaches for surface inspection [17]. Early image-based inspection methods relied on hand-crafted intensity, edge, frequency-domain, and texture features. Tsai et al. [18] used statistical texture analysis and threshold-based segmentation for directional surfaces, while Xie [19] reviewed traditional texture-based defect detection methods. Frustaci et al. [20] presented an in-line machine-vision system for catalytic-converter assembly in which median filtering, Canny edge detection, contour selection, and morphological operations were used to isolate a sensor-boss region before geometric inspection.

These methods can work effectively when imaging conditions are stable and defect shapes are limited. However, in real production lines, strong specular reflection, illumination changes, machining marks, and low defect contrast can occur simultaneously, making the visual difference between defects and normal surfaces unstable. As a result, rule-based methods show limited generalization across various component shapes and defect types.

Subsequent studies shifted toward learning-based defect detection methods using convolutional neural networks (CNNs) to overcome the limitations of hand-crafted feature design. Sun et al. [21] demonstrated improved performance for various metal surface defects using CNN-based feature extraction, and He et al. [22] showed that deep neural networks can learn feature representations that are more robust to defect shape variations. CNN-based methods reduce dependence on manually designed features and can flexibly respond to changes in defect size, shape, and position.

More recently, one-stage object detectors that can simultaneously estimate defect location and class have been widely used in metal surface defect detection. In particular, YOLO-based models are suitable for industrial environments requiring real-time inspection because they provide a good balance between detection speed and accuracy. Zhou et al. [23] improved a YOLOv5-based structure to detect small defects on complex metal surfaces, and Zeng et al. [24] applied a YOLOv8-based model to steel surface defect detection, achieving a balance between real-time processing and detection accuracy. These studies contributed to improving feature representation and localization performance for small defects through detector architecture, feature pyramid, attention modules, and data augmentation.

However, most RGB-based deep learning studies assume that the input image is already aligned around the target object or that a stable single-component ROI has been obtained. In practice, when component positions vary between frames or when background regions are included in the detector input, background texture, illumination reflections, inspection jigs, or rails can become part of the learning and inference targets. Therefore, to maintain the performance of RGB-based defect detectors in real production environments, a preprocessing strategy that physically stabilizes the component-level ROI before defect detection is required in addition to detector architecture improvements.

### 2.2. Sensor-Based Techniques to Aid in Defect Detection

RGB image-based defect detection depends strongly on surface color, texture, and illumination conditions. Therefore, additional sensors have been used to complement inspection inputs in industrial environments. Depth sensors, ToF sensors, and photometric stereo systems can provide distance, shape, surface normals, or reflectance information that is difficult to obtain from RGB images alone, enabling object–background separation, surface structure recovery, and illumination variation mitigation.

Xu et al. [25] combined depth information with RGB images for steel surface defect inspection and attempted to reduce position uncertainty in RGB-only inspection by using depth information to estimate object shape and location. Saiz et al. [26] used a photometric stereo-based system for inspecting metallic components with strong reflective properties. By combining images acquired under multiple illumination conditions, their method recovered surface normals and reduced highlight and shadow distortions that occur under single-light conditions. Wang et al. [27] proposed CLANet, which uses both RGB and depth images for surface defect inspection of rails. Their method uses depth information as complementary spatial information to reduce false detections caused by visual similarity between the inspection target and the background.

These studies commonly use additional sensor information to complement RGB-based defect detection inputs or to separate objects from backgrounds more stably. However, for metallic surfaces, strong specular reflection can make depth measurements unstable or incomplete, and ToF intensity images can exhibit repeated signal attenuation or dropout patterns. Previous studies have often regarded these phenomena as sensor noise, depth artifacts, or unstable signals requiring correction. In contrast, ToF intensity images directly reflect the spatial variation in received sensor signals and the reflectance characteristics of metal surfaces. Although they may not represent the defect itself precisely, they can provide useful cues for estimating the spatial support of the component.

### 2.3. Research Gap and Our Approach

Taken together, RGB-based deep learning approaches are effective for learning visual features of fine defects and estimating their location and class. However, their detection stability can deteriorate when irrelevant background regions are included in the detector input or when component positions vary between frames. Sensor-based approaches such as depth, ToF, and photometric stereo are useful for object–background separation or surface structure estimation, but their measurements can be unstable on highly reflective metal surfaces, and they are not always suitable for directly detecting fine defects.

To address this gap, this study uses ToF intensity signals not as direct inputs for defect detection but as localization cues for stabilizing component-level ROIs before detection. The ToF intensity dropout patterns caused by specular reflection on metallic surfaces are reinterpreted as structural signals for extracting foreground support, and these signals are used to restrict the analysis region of the RGB detector to the component surface. Specifically, the proposed method separates foreground support regions extracted from the ToF intensity image into individual object candidates using connected-component analysis and directly projects them onto the RGB coordinate system using a pre-calibrated fixed homography. The spatial extent of the projected foreground support is then used to generate an object-adaptive bounding box, which serves as the final RGB ROI. This process does not require frame-by-frame feature matching or complex multimodal feature fusion, and it reconfigures the detector input distribution around the component without modifying the RGB detector architecture. Thus, the proposed method combines the defect representation capability of RGB detectors with the object localization stability of ToF intensity information, providing a lightweight cross-modal ROI generation framework that mitigates background inclusion and position instability in metal surface defect detection.

Accordingly, the two sensing modalities are assigned different roles in the proposed system: ToF intensity provides the component location, while the RGB image is used for defect detection. This arrangement allows the ROI to be stabilized without modifying the detector itself.

## 3. Methodology

This section describes the overall structure and operation of the proposed ToF-guided object-adaptive ROI generation framework. The proposed method first extracts foreground support from the ToF intensity image, aligns it with the RGB coordinate system, and generates component-level ROIs for defect detection. The generated RGB ROI is then provided to a YOLO-based defect detector to predict the location and class of metal surface defects.

### 3.1. Overall Framework

**Figure 1:**
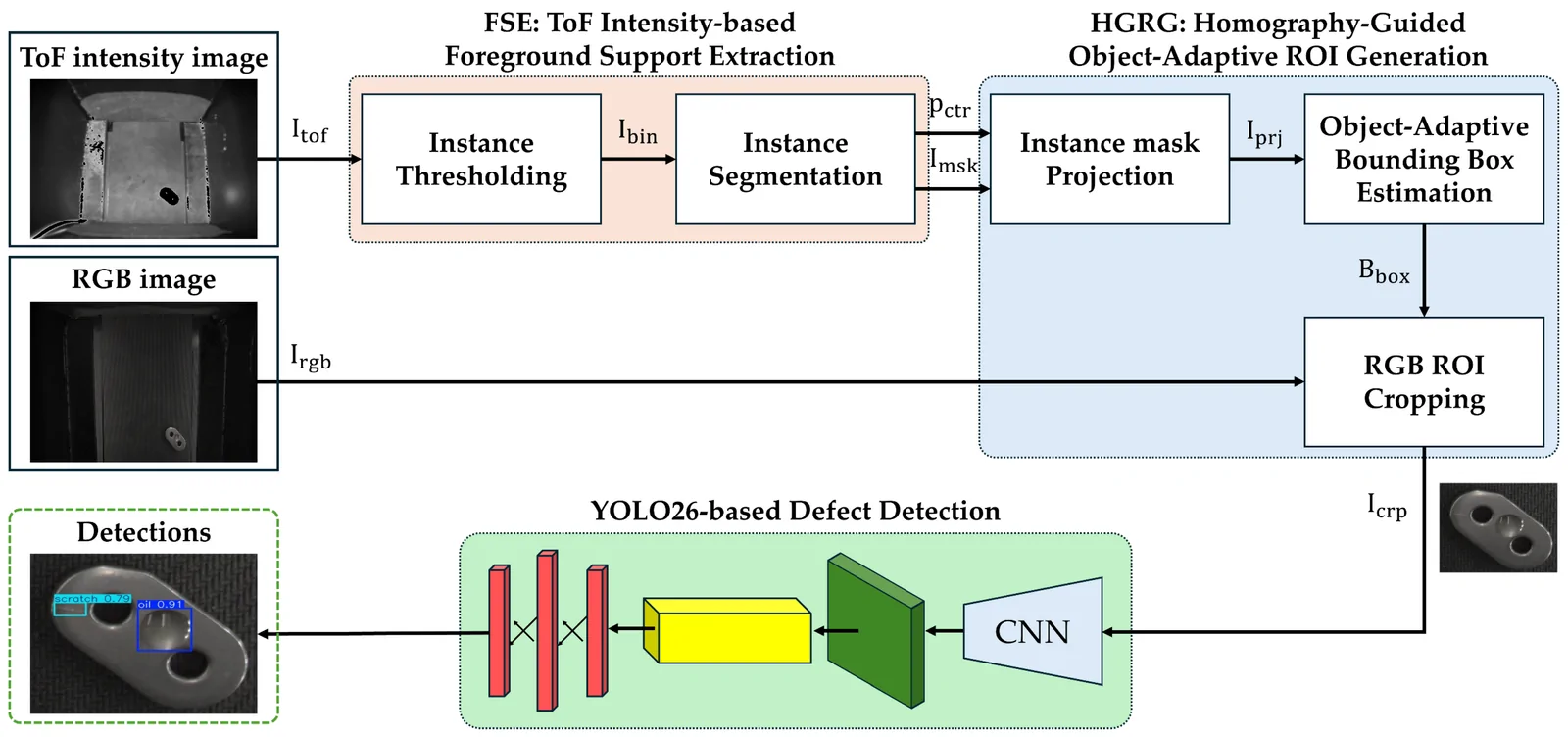
Overall pipeline of the proposed ToF-guided object-adaptive ROI generation framework.

The overall pipeline of the proposed method is shown in Figure 1. The framework consists of ToF intensity-based foreground support extraction (FSE), homography-guided object-adaptive ROI generation (HGRG), and YOLO-based defect detection. First, an RGB image $I_{rgb}$ and a ToF intensity image $I_{tof}$ are simultaneously acquired from the RGB camera and the ToF sensor. From $I_{tof}$, foreground support regions are extracted using intensity attenuation or dropout patterns caused by specular reflection on the metal surface. These foreground support regions are then separated into individual object candidates through connected-component analysis. For each valid candidate, the foreground support pixels and their centroid are projected onto the RGB coordinate system using a fixed homography matrix obtained through prior calibration. Based on the spatial extent of the projected support region, an object-adaptive bounding box is generated and used to crop the RGB image into a component-level ROI. This process centers the detector input around the target component even when the component position varies, while reducing the inclusion of irrelevant background texture and reflection patterns. Finally, the cropped RGB ROI is provided to a YOLO-based defect detector for surface defect detection. 

### 3.2. ToF Intensity-Based Foreground Support Extraction

ToF intensity-based foreground support extraction is the stage that extracts the foreground support of a metal component from the ToF intensity image and separates it into individual object candidates. Owing to the high reflectance and specular reflection characteristics of metallic surfaces, the IR signal received by the ToF sensor can be saturated or weakened in some regions. As a result, low-intensity responses or dropout patterns repeatedly appear in the ToF intensity image. Although such regions may be regarded as sensor noise or unstable measurements, this study focuses on their potential use as structural signals that reflect the surface shape and spatial location of metal components.

The input ToF intensity image $I_{tof}$ is given as a grayscale image, and foreground support is extracted using a predefined threshold $T_{bin}$. Let $R^{H\times W}$ denote the set of all image pixel coordinates. The binary mask $I_{bin}$ is defined for each pixel position $s\in R^{H\times W}$ as follows.

(1) $$I_{bin}\left(s\right)=1\left(I_{tof}\left(s\right)<T_{bin}\right)$$

Here, 1(⋅) is an indicator function that returns 1 if the condition is true and 0 otherwise. Pixels with $I_{bin}\left(s\right)=1$ are defined as foreground support pixels with low ToF intensity responses, and the set of foreground pixels is denoted by $F=\left\{s\right|I_{bin}\left(s\right)=1\}$. Connected component labeling (CCL) is then applied to separate the foreground pixels into individual object candidates. This study uses 8-neighborhood connectivity. For two foreground pixels $p=\left(x_{p},y_{p}\right)$ and $q=\left(x_{q},y_{q}\right)$, the 8-neighborhood relation is defined as follows.

(2) $$N\left(p\right)=\{q\in F|\max\left(\left|x_{p}-x_{q}\right|,\left|y_{p}-y_{q}\right|\right)=1\}$$

If two foreground pixels p,q∈F are reachable through a path composed only of foreground pixels under the 8-neighborhood relation, they are considered to belong to the same connected component. Accordingly, the k-th connected component $C_{k}$ is represented as follows.

(3) $$C_{k}=\left\{q\in F\right|q∼p_{k}\}$$

Here, $p_{k}$ denotes an arbitrary seed foreground pixel belonging to the k-th connected component, and $q∼p_{k}$ indicates that q is connected to $p_{k}$ through an 8-connected foreground path. However, owing to the reflective characteristics of the ToF sensor, background reflections, or measurement noise, foreground pixels may also be detected outside the actual inspection region. To remove such irrelevant foreground responses, the centroid of each connected component is calculated as the arithmetic mean of its constituent pixel coordinates. The geometric center $p_{geo,k}$ of the k-th connected component is calculated as follows.

(4) $$p_{geo,k}=\left(\frac{1}{\left|C_{k}\right|}\sum_{p\in C_{k}}x_{p},\frac{1}{\left|C_{k}\right|}\sum_{p\in C_{k}}y_{p}\right)$$

To determine whether the computed centroid lies within the valid inspection region, a ray casting-based point-in-polygon test is applied. Let $\{E_{i}{\}}_{i=1}^{M}$ denote the set of polygon edges composing the predefined inspection region $R_{ins}$, and let $r\left(t\right)=p_{geo,k}+t\left(1,0\right),t\geq 0$, be a ray extending from $p_{geo,k}$ in the positive x-direction. The number of intersections $n\left(p_{geo,k}\right)$ is computed as follows.

(5) $$n\left(p_{geo,k}\right)=\sum_{i=1}^{M}1\left(r\left(t\right)\cap E_{i}\neq \emptyset \right)$$

If the number of intersections is odd, the centroid is determined to be inside the inspection region.

(6) $$p_{geo,k}\in R_{ins}⟺n\left(p_{geo,k}\right)\equiv 1\left(mod\;2\right)$$

Small noisy regions are removed by excluding components whose number of pixels $\left|C_{k}\right|$ is smaller than a predefined area threshold $T_{area}$. For all experiments reported here, the intensity threshold was set to 100 and the minimum connected-component area to 1200 pixels. Finally, only connected components satisfying both the area condition and the ROI inclusion condition are retained as valid object candidates. The center coordinate $p_{ctr,k}$ of the valid object candidate is defined as follows.

(7) $$p_{ctr,k}=\{\begin{array}{ll} p_{geo,k}, & \text{if}\;\left|C_{k}\right|\geq T_{area}\;\text{and}\;p_{geo,k}\in R_{ins}\\ \text{undefined}, & \text{otherwise} \end{array}$$

This filtering process removes connected components generated by background reflections or irrelevant intensity responses outside the inspection region. For each valid object candidate, the connected component $C_{k}$ associated with $p_{ctr,k}$ is converted into an instance mask as follows.

(8) $$I_{msk,k}\left(p\right)=\{\begin{array}{c} 1,\;\;\text{if}\;p\in C_{k}\;\text{and}\;p_{ctr,k}\neq \text{undefined}\\ 0,\;\;\text{otherwise} \end{array}$$

Through this process, the ToF intensity image is used to extract object-related foreground support and generate instance-level masks after removing noise components outside the inspection region. The generated instance mask $I_{msk,k}$ is then used in the next stage for homography-guided object-adaptive ROI generation.

### 3.3. Homography-Guided Object-Adaptive ROI Generation

Homography-guided object-adaptive ROI generation aligns the foreground support extracted from the ToF intensity image with the RGB image coordinate system and generates RGB ROIs for defect detection. This stage consists of cross-modal homography calibration, foreground projection, object-adaptive bounding box estimation, and ROI cropping.

#### 3.3.1. Cross-Modal Homography Calibration

A homography matrix H is estimated to define the spatial correspondence between the ToF image coordinate system and the RGB image coordinate system. In the experimental setup, the RGB camera and ToF camera have a fixed geometric configuration, and metal components move within a limited inspection region. Because the goal of this study is not pixel-level 3D reconstruction but the alignment of object support regions for defect detection, the correspondence between the two sensors is approximated by a single-plane projective transformation. This approximation is valid under the condition that the height variation in the component is sufficiently small relative to the camera–object distance and the ROI padding margin.

During calibration, corresponding points in the ToF and RGB coordinate systems are manually specified from the same scene. Let $p_{tof,i}=\left(x_{i},y_{i}\right)$ and $p_{rgb,i}=\left({x_{i}}^{\prime },{y_{i}}^{\prime }\right)$ denote the i-th corresponding point pair. A set of N visually identifiable point correspondences, where N≥4, is used to estimate the homography. To reduce the influence of manual annotation errors and outliers, random sample consensus (RANSAC) is applied. For each candidate homography H, the reprojection error $e_{i}\left(H\right)$ of the i-th correspondence is computed as follows.

(9) $$e_{i}\left(H\right)=\|p_{rgb,i}-\pi ({H{\tilde{p}}_{tof,i})\|}_{2}$$

Here, ${\tilde{p}}_{tof,i}={\left(x_{i},y_{i},1\right)}^{⊤}$ denotes the homogeneous coordinate of the ToF point, and π(⋅) is the projection operator that converts homogeneous coordinates into 2D coordinates by normalization.

RANSAC randomly samples four point pairs to estimate a candidate homography and evaluates the number of inliers whose reprojection errors are smaller than a predefined threshold $T_{rep}$. The optimal homography $H_{opt}$ is then defined as the one that maximizes the number of inliers.

(10) $$H_{opt}=\underset{H}{\mathrm{argmax}}\sum_{i=1}^{N}1\left(e_{i}\left(H\right)<T_{rep}\right)$$

The estimated $H_{opt}$ is stored as a static parameter. During online inspection, no additional feature matching or homography re-estimation is performed for each frame. Instead, ToF foreground pixels are directly projected onto the RGB coordinate system using the stored $H_{opt}$. Although homography-based alignment cannot fully compensate for all 3D shape variations, a fixed padding margin is added to the final ROI to mitigate projection errors caused by component height differences and planar approximation.

#### 3.3.2. Foreground Projection and ROI Cropping

For the k-th valid object candidate extracted from the ToF intensity image, the foreground pixels in $C_{k}$ are projected onto the RGB coordinate system using the optimized homography matrix $H_{opt}$. For a point p=(x,y) in the ToF coordinate system, its corresponding projected point $p^{\prime }=\left(x^{\prime },y^{\prime }\right)$ in the RGB coordinate system is computed as follows.

(11) $$\lambda \left[\begin{array}{c} x^{\prime }\\ y^{\prime }\\ 1 \end{array}\right]=H_{opt}\left[\begin{array}{c} x\\ y\\ 1 \end{array}\right]$$

Here, λ denotes the homogeneous scale factor. This projection is applied to both the centroid and the foreground support pixels. When $p\in C_{k}$, the projected points form the foreground pixel set $C_{k}^{\prime }$ in the RGB coordinate system. Instead of transforming the entire ToF image, only the foreground support pixels of each valid object candidate are projected. This reduces unnecessary background transformations and enables ROI generation around the object support region.

The projected foreground pixels are represented as a binary mask in the RGB coordinate system. If $I_{prj,k}$ is defined as the projected mask of the k-th object candidate, it can be expressed as follows.

(12) $$I_{prj,k}(p^{\prime })=\{\begin{array}{ll} 1, & \text{if}\;p^{\prime }\in C_{k}^{\prime }\\ 0, & \text{otherwise} \end{array}$$

The object-adaptive bounding box $B_{box,k}$ is then generated from the spatial extent of the projected foreground pixel set $C_{k}^{\prime }$. It is defined as follows.

(13) $$B_{box,k}=\left[x_{min}-m,\;y_{min}-m,\;x_{max}+m,\;y_{max}+m\right]$$

Here, $x_{min}$, $y_{min}$, $x_{max}$, and $y_{max}$ are the minimum and maximum coordinates of the projected pixels in $C_{k}^{\prime }$ and m is the ROI padding margin. The padding margin was set to 5 pixels in all experiments. Because the ToF foreground response may appear sparsely in only part of the object depending on the reflective state of the metal surface, the padding margin ensures that the final RGB ROI includes the entire component surface and possible defect regions while minimizing background inclusion. Before ROI extraction, the generated bounding box $B_{box,k}$ is clamped to the valid RGB image boundaries to prevent invalid crop coordinates. The final RGB ROI $I_{crp,k}$ is then cropped from the original RGB image $I_{rgb}$ as follows.

(14) $$I_{crp,k}=I_{rgb}\left[{x_{min}-m:x_{max}+m,\;\;y}_{min}-m:y_{max}+m\right]$$

**Figure 2:**
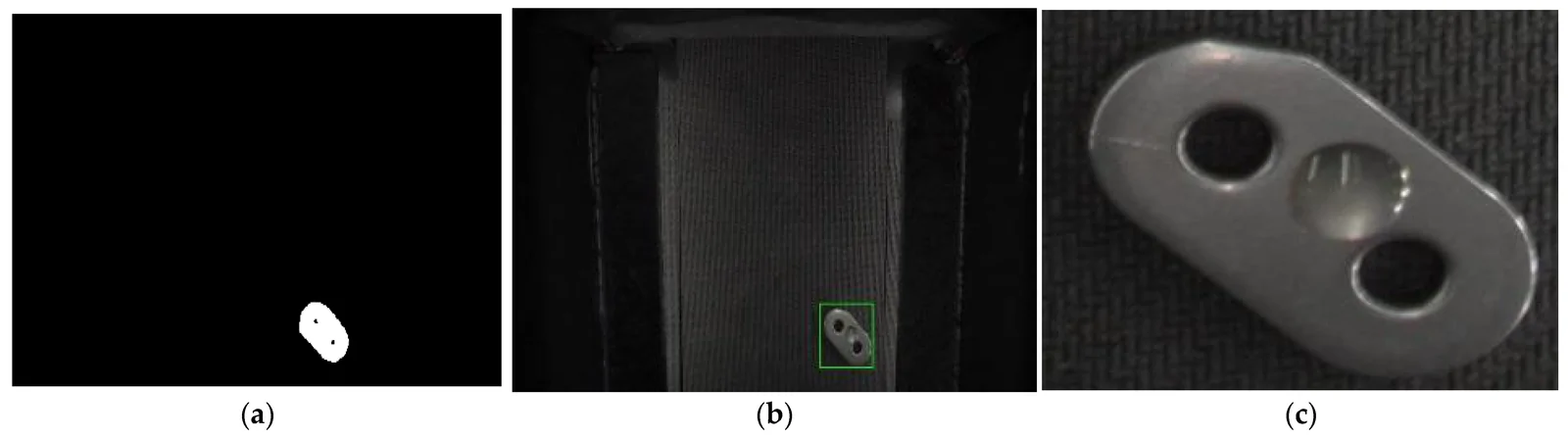
Example results of homography-guided object-adaptive ROI generation: (**a**) instance mask generated by FSE, (**b**) foreground support region projected onto the RGB coordinate system, and (**c**) cropped RGB ROI.

If no foreground pixel is projected within the valid RGB image region, the corresponding object candidate is discarded. If valid projected foreground pixels exist, the cropped RGB ROI is used as the detector input. As a result, the proposed ROI generation process produces consistent object-centered RGB ROIs even when component positions vary, thereby reducing the inclusion of background texture and reflection patterns in the detector input. As a result, the proposed ROI generation process produces consistent object-centered RGB ROIs even when component positions vary and reduces the inclusion of background texture and reflection patterns in the detector input. Figure 2 illustrates representative results of the proposed homography-guided object-adaptive ROI generation process.

### 3.4. YOLO-Based Defect Detection

**Figure 3:**
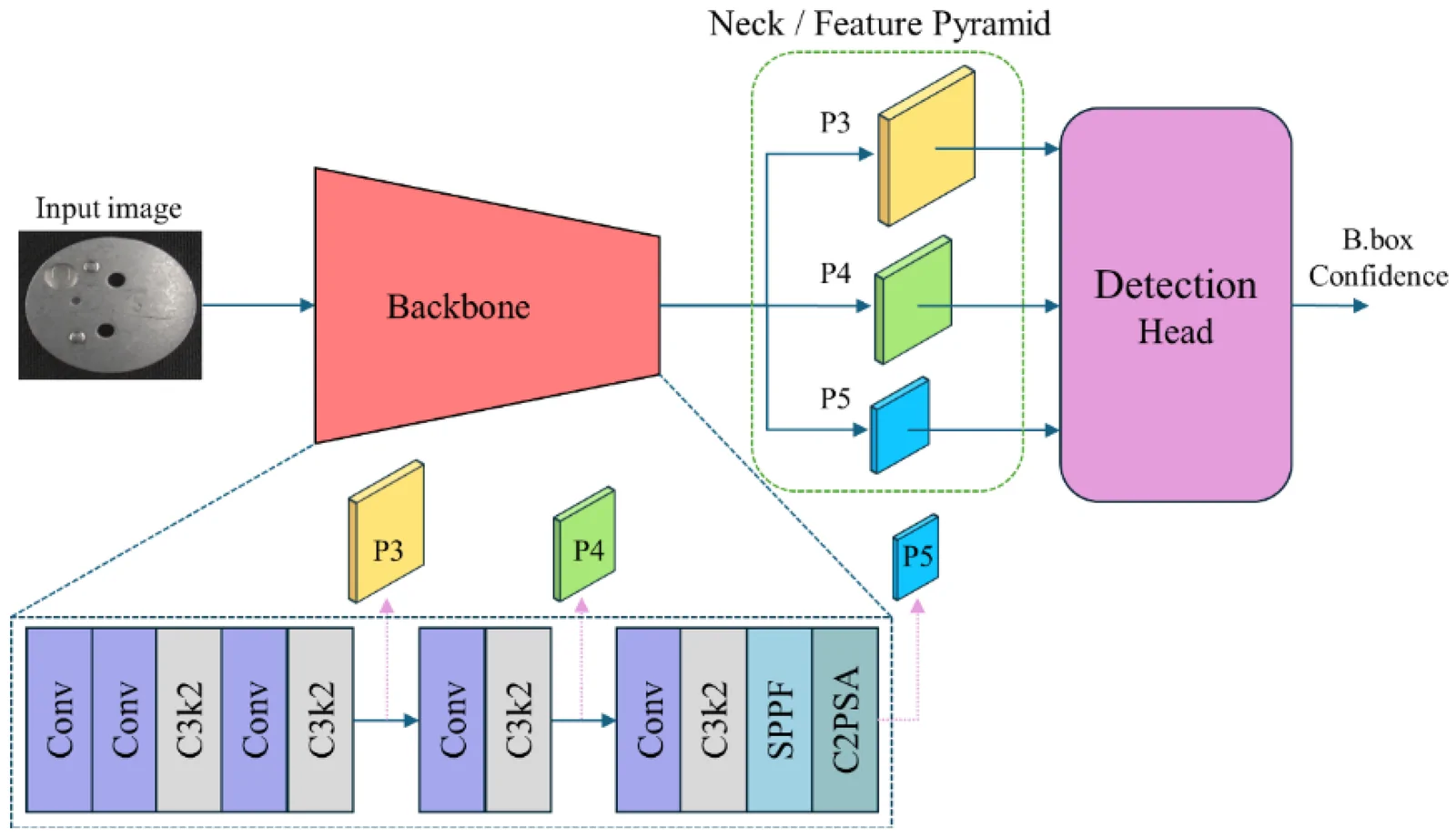
Schematic illustration of the YOLO26-based defect detection architecture.

This study detects metal surface defects using the RGB ROIs generated by homography-guided object-adaptive ROI generation. YOLO26 is used as the object detection model. Figure 3 shows the schematic architecture of the YOLO26-based defect detection model used in this study. YOLO26 is a YOLO-series model designed for real-time object detection, and it reduces dependence on postprocessing through an end-to-end detection structure and NMS-free inference. It also considers model efficiency and small-object detection stability through design elements such as Distribution Focal Loss removal, ProgLoss, and Small-Target-Aware Label Assignment. These characteristics are suitable for detecting metal surface defects that are small and have unclear boundaries.

The detection head of YOLO26 predicts class probabilities and bounding box coordinates for each defect candidate in the input ROI. During training, the model jointly minimizes classification loss and bounding box regression loss to improve both defect class classification and localization accuracy. The total loss can be generally expressed as follows.

(15) $$L_{total}=\lambda_{cls}\cdot L_{cls}+\lambda_{box}\cdot L_{box}$$

Here, $L_{cls}$ and $L_{box}$ denote the classification loss and bounding box regression loss, respectively, while $\lambda_{cls}$ and $\lambda_{box}$ control the relative contributions of the two terms. In a simplified class-wise binary cross-entropy form, the classification loss can be written as follows.

(16) $$L_{cls}=-\sum_{c=1}^{CL}\alpha_{c}\log\left(\beta_{c}\right)+\left(1-\alpha_{c}\right)\log\left(1-\beta_{c}\right)]$$

where, CL is the number of defect classes, $\alpha_{c}\in \left\{0,1\right\}$ is the ground-truth label for class c, and $\beta_{c}\in \left[0,1\right]$ is the predicted class confidence. The bounding box regression loss increases the spatial agreement between predicted and ground-truth bounding boxes and can be written in simplified form as follows.

(17) $$L_{box}\propto 1-IoU\left(B_{pred},B_{gt}\right)$$

Here, IoU denotes intersection over union, and $B_{pred}$ and $B_{gt}$ denote the predicted and ground-truth bounding boxes, respectively. The proposed ROI generation does not modify the internal structure or training objective of YOLO. Instead, it reduces the proportion of background texture and reflection patterns in the detector input and increases the relative proportion of defect candidate regions. Thus, the classifier learns class confidence from inputs with reduced background-defect confusion, while box regression learns defect locations within more consistently aligned component-centered ROIs.

## 4. Experimental Results

This section evaluates the proposed framework using an RGB-ToF dataset constructed under conditions similar to practical industrial inspection settings. The experiments are designed with three objectives. First, a suitable baseline detector for the dataset is selected. Second, the effects of full-image input, sliding-window input, and the proposed ToF-guided object-adaptive ROI input are compared using the same detector. Third, the proposed FSE- and HGRG-based ROI generation framework is compared quantitatively with existing object detection and preprocessing-based methods. Performance is evaluated using Precision (TP/(TP+FP)), Recall (TP/(TP+FN)), mAP@50, mAP@50–95 [28], and average processing time per image; TP is true positive, FP is false positive, and FN is false negative.

### 4.1. Experimental Setup

To evaluate the proposed framework, a custom RGB-ToF dataset was constructed using defective metal components. Images were acquired using a Basler Ace2 RGB camera (Basler AG, Ahrensburg, Germany) [29] and a LUCID Vision Helios2 ToF camera (LUCID Vision Labs, Inc., Richmond, BC, Canada) [30]. A Ricoh FL-CC0614A-2M lens (Rocoh Co., Ltd., Tokyo, Japan; F1.4, f = 6 mm, 2/3 inch) [31] was mounted on the RGB camera. RGB images were acquired at a resolution of 3840×2160 and used for defect detection, while ToF images were acquired at a resolution of 640×480 and used as auxiliary information for object localization and ROI generation. The two sensors were fixed inside the same housing to simultaneously acquire paired RGB and ToF intensity images.

The dataset used in this study was not derived from a public benchmark. The imaged specimens were actual industrial metal components classified as normal or defective during the manufacturing process, and the acquisition chamber was configured to reproduce controlled optical inspection conditions relevant to the target industrial application. Therefore, the evaluation reflects real manufactured parts and manufacturing-related defect characteristics, while remaining a controlled experimental setup rather than an on-line production-line deployment.

**Figure 4:**
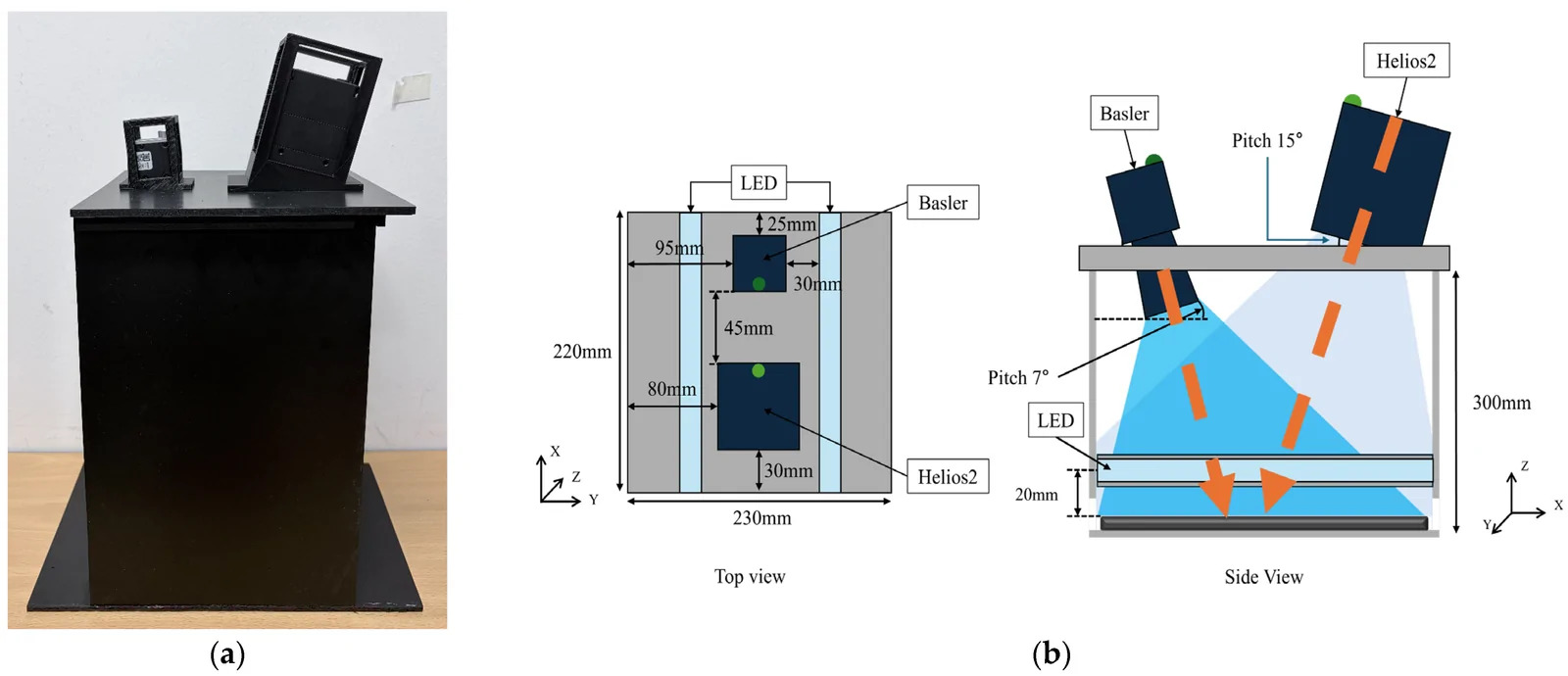
Image acquisition chamber used for RGB-ToF data collection: (**a**) external view and (**b**) internal component arrangement.

Component images were captured inside a light-shielding chamber to minimize the influence of external illumination. The external appearance and internal configuration of the chamber are shown in Figure 4. The chamber was constructed using black foam board, and a 5 mm-thick rubber sheet was installed in the rail region where components moved to suppress unnecessary light reflections. LED strips with a color temperature of 6000 K were placed at the top and bottom of the chamber to provide a consistent illumination environment. This configuration provides sufficient illumination for observing metal surface defects in RGB images while reducing the effects of external lighting variations. The illumination level was set to approximately 150 lux, following the inspection condition specified by the collaborating industrial partner.

**Figure 5:**
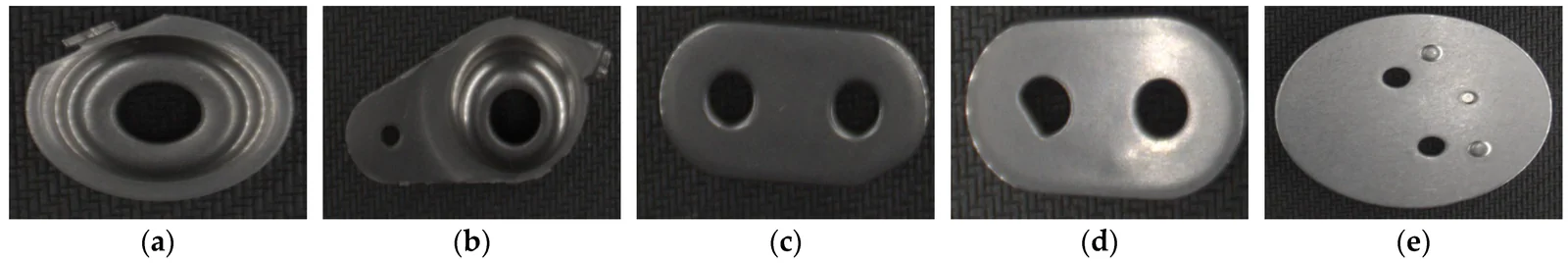
Metal components used in the experiments: (**a**) Cap-1, (**b**) Cap-2, (**c**) Clip-8, (**d**) Clip-V, (**e**) Clip-O.

A total of 775 RGB-ToF image pairs were used in this study. The dataset consists of 775 RGB images and 775 corresponding ToF images, each acquired at the same time. The ToF image was an infrared intensity image acquired in luminance mode. The metal components used for imaging were actual parts classified as normal or defective in the manufacturing process, with diameters ranging from 25 mm to 48 mm. The dataset contains five component types, and for each component type, normal, contamination, and damage states were captured. The training set consists of 625 image pairs: 25 normal, 50 contamination, and 50 damage samples for each component type. The evaluation set consists of 150 image pairs: 5 normal, 10 contamination, and 15 damage samples for each component type. Each RGB-ToF pair corresponds to a different physical component, and no component is shared between the training and evaluation sets. Figure 5 shows examples of the components used in the experiments.

**Figure 6:**
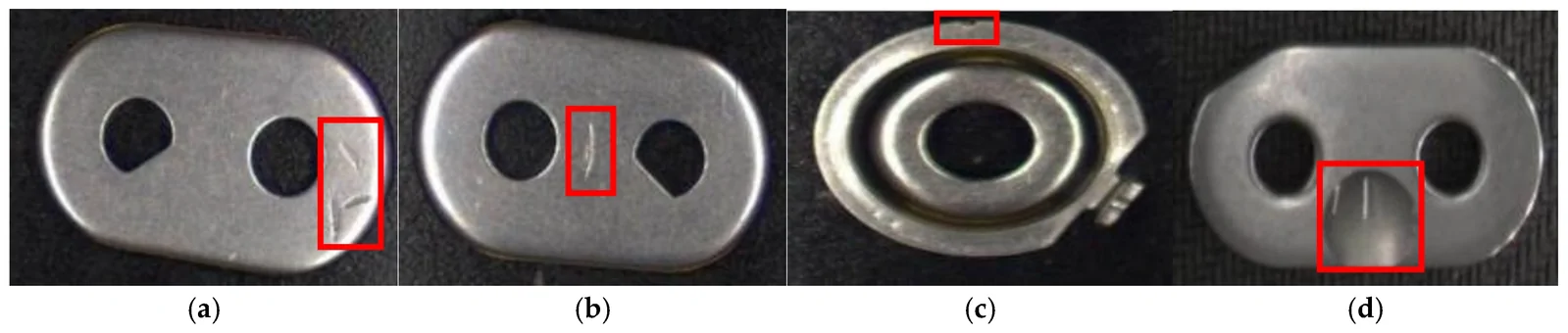
Representative defect types in the constructed dataset: (**a**) Nick, (**b**) Dent, (**c**) Scratch, and (**d**) Oil contamination.

The constructed dataset includes various surface defects that frequently occur in metal component production lines. Representative defect examples are shown in Figure 6. Nick refers to local indentation caused by physical impact, dent refers to deformation of the edge or surface, scratch refers to a surface scratch, and oil refers to oil contamination. The red boxes indicate the defect regions.

The objective of this study is not to classify detailed defect subtypes, but to improve overall defect detection performance by stably generating component-level ROIs before detection. Therefore, Nick, Dent, and Scratch were integrated into the Damage class, whereas Oil was defined as the Contamination class. The final defect detection problem thus consists of two defect classes: Contamination and Damage. Each image contains one randomly selected component within the predefined inspection region, consistent with the target inspection process considered in this study. Multi-component or overlapping-component scenarios are left for future work. All experiments were performed on a Windows 11 workstation equipped with an Intel i9-10900X CPU at 3.70 GHz and an NVIDIA TITAN RTX GPU with 24 GB VRAM. All models used the same training and evaluation split, and the input resolution, batch size, and number of epochs were kept identical for fair comparison.

### 4.2. Ablation Study

To analyze the effect of the proposed FSE- and HGRG-based ROI generation framework, a series of ablation studies was performed. First, Gold-YOLO, YOLO11, and YOLO26 were compared to select a suitable baseline detector for the dataset. Table 1 shows the performance of each detector when the full RGB image is used as input. 

Gold-YOLO [30], YOLO11 [32], and YOLO26 [33] were evaluated on the same dataset. The results show that a complex feature fusion structure does not always lead to higher defect detection performance for this dataset. Unlike general object detection datasets, the defect objects used in this study are small, have low intensity contrast with the metal surface, and are visually similar to background texture and reflection patterns. Therefore, detection performance is strongly influenced by the ability to distinguish local intensity variations and subtle texture differences around defect boundaries rather than by global semantic representation. Gold-YOLO repeatedly fuses multi-scale features; however, under the conditions of this dataset, defect and background features may be mixed, weakening the local features of small defects. By contrast, YOLO11 and YOLO26 maintained fast processing speed and stable detection performance through relatively lightweight detector structures. In particular, YOLO26 achieved higher Precision and mAP@50 than YOLO11 while maintaining the same processing time. Therefore, YOLO26 was selected as the baseline detector for subsequent experiments.

Next, the effect of input ROI strategy was evaluated using the same YOLO26 detector. Table 2 compares full-image input, SAHI-based sliding-window input, and the proposed ToF-guided object-adaptive ROI input. This experiment isolates the effect of input configuration while keeping the detector architecture fixed.

When the full image is used as input, YOLO26 performs detection based on global information without any ROI generation step and therefore shows the fastest processing speed. However, metal surface defects are thin, irregularly shaped, and have low intensity contrast with the background. Consequently, defect features may be diluted by surrounding texture and reflection patterns in full-image inputs, limiting the ability of the detector to distinguish defects from background patterns. SAHI [35] divides the input image into overlapping patches to improve small-object detection. Although this strategy can be effective for general small-object detection, the defects in this study are not only small but also low-contrast patterns with unclear boundaries and texture-based features. Because sliding-window slicing does not guarantee object-centered alignment, background texture may be emphasized as a defect candidate in some patches, increasing false positives. In addition, repeated inference over many patches substantially increases processing time, limiting its suitability for real-time inspection systems.

In contrast, the proposed method projects the foreground support extracted from the ToF intensity image onto the RGB coordinate system and generates a component-centered object-adaptive ROI. This strategy is different from simple RGB intensity-based cropping because it uses ToF intensity information to reconfigure the detector input around the actual component region. As a result, background reflections and irrelevant texture information are reduced, and the relative proportion of defect candidate regions within the input ROI increases. In Table 2, the proposed method achieved Precision of 0.828, Recall of 0.745, and mAP@50 of 0.796, outperforming both full-image input and SAHI-based input. These results demonstrate that the proposed ROI strategy reconfigures the detector input distribution into a form favorable for defect detection.

To evaluate repeated-run stability, five experiments were conducted using different random seeds. Table 3 shows the repeated-run results of the baseline YOLO26 and the proposed method. The proposed method achieved a mean mAP@50 of 0.796 with a standard deviation of 0.013, which is higher than the mean mAP@50 of 0.585 obtained by the baseline YOLO26. The standard deviation was also comparable to that of the baseline. These results indicate that the proposed ToF-guided ROI generation provides stable performance improvement without substantially increasing variation caused by random initialization. In particular, FSE and HGRG reduce irrelevant background regions and construct component-centered detector inputs, allowing the detector to respond more consistently to local defect features.

### 4.3. Quantitative Comparison

To comprehensively evaluate the proposed framework, comparative experiments were performed with YOLO26 [34], YOLO26+SAHI [35], RT-DETR [36], DEIM [37], and AIE and ESE [38]. These methods were selected to represent different detection structures and input processing strategies. YOLO26 served as the baseline detector using full RGB images, RT-DETR represented a transformer-based detector with global attention, DEIM represented an improved matching-based real-time DETR detector, AIE and ESE represented a preprocessing-based method combining illumination enhancement and edge feature extraction, and YOLO26+SAHI represented a slicing-based inference strategy for small-object detection. All models were evaluated using the same defect class definitions and evaluation metrics. For fair comparison, the input image resolution was set to 640×640, the batch size was set to 16, and all models were trained for 150 epochs. Processing time was calculated as the average per-image time including input preprocessing, ROI generation or slicing, and detector inference.

Table 4 presents the quantitative comparison between the proposed framework and existing object detection or preprocessing-based methods. YOLO26 [33] provided fast processing speed and balanced detection performance, but it uses the full RGB image as input and therefore includes unnecessary background regions and metal reflection patterns. Such background texture can resemble defects, increasing false positives and localization instability. SAHI [35] is a sliding-window-based method that is effective for small objects, but it showed low Precision and Recall on this dataset. This is because patch-wise slicing can separate continuous defect shapes from their surrounding context and may emphasize background texture more than actual defects in some patches. Repeated patch inference also increased processing time to 3642 ms/image, limiting real-time applicability. RT-DETR [36] can capture global context through its transformer-based attention structure. However, the defects in this study are small and appear as low-contrast texture patterns; thus, local boundary and intensity variations are more important than global context. As a result, RT-DETR showed limited Recall and mAP for some fine defects. DEIM [37] achieved relatively high Precision but low Recall, indicating that some defect candidates in low-contrast and irregular defect regions were not sufficiently converted into positive predictions. AIE and ESE [38] improved Precision by enhancing input contrast and edge information, but it did not directly address object–region alignment or background inclusion, resulting in limited Recall and mAP@50–95 performance.

By contrast, the proposed method effectively reduced background inclusion by generating component-centered ROIs through ToF intensity-based foreground support extraction and homography-guided RGB projection. Consequently, the relative proportion of defect candidate regions in the detector input increased, and the local features of low-contrast and small defects were preserved more stably. The proposed method achieved the best performance across all evaluation metrics, with Precision of 0.828, Recall of 0.745, mAP@50 of 0.796, and mAP@50–95 of 0.517. Compared with the baseline YOLO26, mAP@50 improved from 0.585 to 0.796, and mAP@50–95 improved from 0.350 to 0.517. These results show that the proposed object-adaptive ROI generation improves localization stability not only under a low IoU condition but also across multiple IoU thresholds.

**Figure 7:**
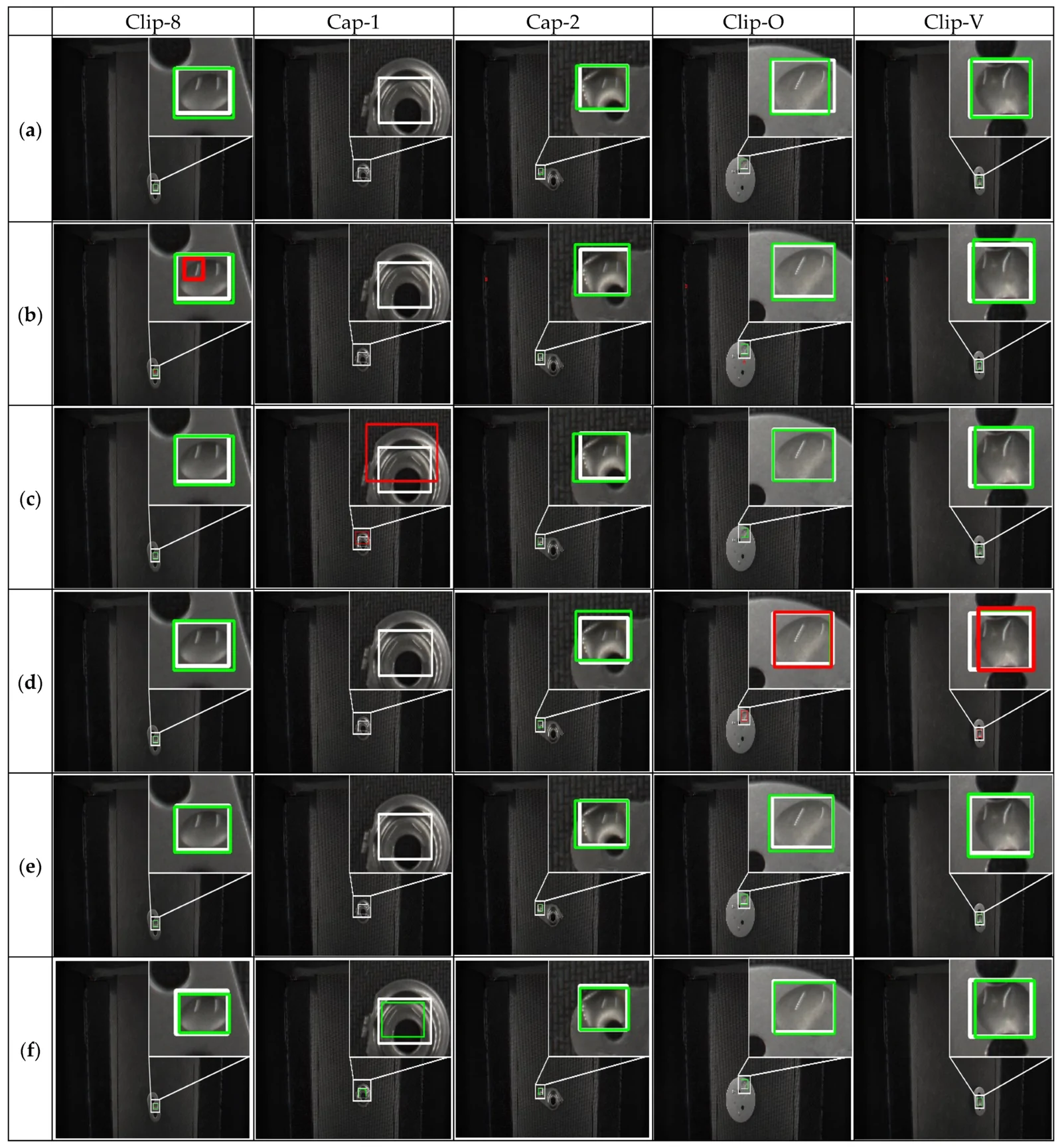
Qualitative detection results for the Contamination class: (**a**) YOLO26, (**b**) YOLO26 + SAHI, (**c**) RT-DETR, (**d**) DEIM, (**e**) AIE and ESE, and (**f**) proposed method.

**Figure 8:**
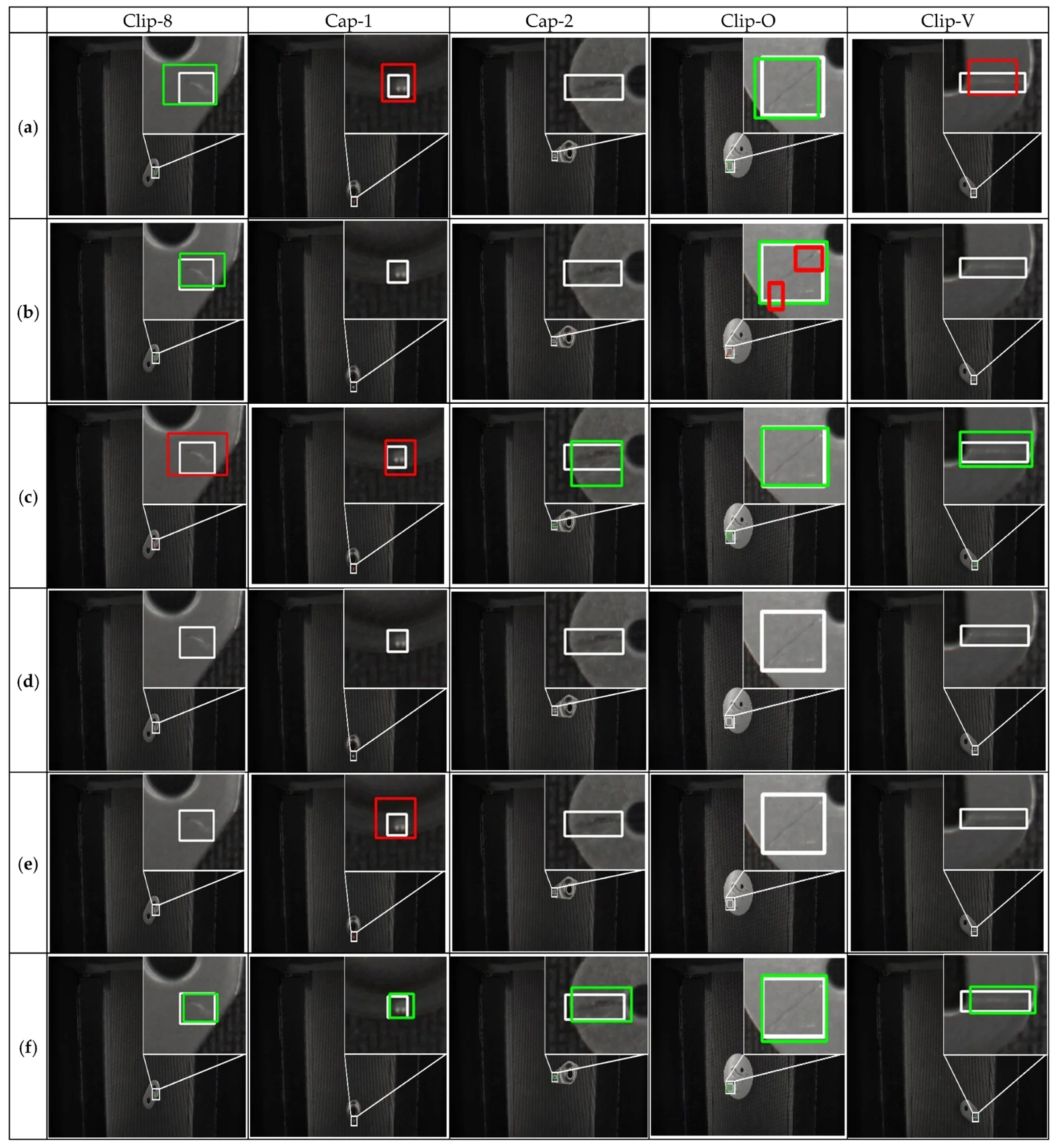
Qualitative detection results for the Damage class: (**a**) YOLO26, (**b**) SAHI, (**c**) RT-DETR, (**d**) DEIM, (**e**) AIE and ESE, and (**f**) proposed method.

Figure 7 and Figure 8 show qualitative detection results for the Contamination and Damage classes, respectively. In the images, white boxes indicate ground truth, green boxes indicate true positives, and red boxes indicate false positives. All results were visualized using a confidence threshold of 0.5. For the Contamination class in Figure 7, most models detected relatively clear contamination regions to some extent. However, when metallic reflection patterns visually resembled contamination defects, some existing models produced false positives or missed detections. In particular, SAHI tended to misclassify background texture as defects in some patches because object-centered alignment was not guaranteed by uniform patch slicing. In contrast, the proposed method detected defect candidates within component-centered ROIs, reducing background interference and providing more stable localization for contamination regions.

The effect of the proposed method was more evident for the Damage class in Figure 8. Damage defects were often long, thin, or irregular and had lower contrast than contamination, making localization unstable for full-image-based detectors. Some existing models repeatedly missed damage regions, and SAHI produced false positives owing to patch boundaries and background texture. In contrast, the proposed method produced stable localization results for defect regions of various shapes and sizes, generating bounding boxes that were well aligned with the ground truth. Even for thin and irregular damage defects, the proposed method stably included defect regions and reduced both false positives and false negatives compared with existing methods.

Overall, both quantitative and qualitative results indicate that stabilizing the input ROI around the component before defect detection is more important for metal surface defect detection than simply increasing detector complexity. In particular, the proposed FSE- and HGRG-based preprocessing framework mitigates background inclusion and position instability without modifying the detector structure and improves detection stability for low-contrast and irregular defects.

## 5. Discussion

The results indicate that the main benefit of the proposed framework comes from stabilizing the detector input before inference. With the same YOLO26 detector, the ToF-guided ROI increased mAP@50 from 0.585 to 0.796 and improved both Precision and Recall (Table 2). Similar gains were observed against the other detection and preprocessing methods in Table 4. These comparisons show that, for the present dataset, reducing irrelevant background content before detection is effective without changing the detector architecture.

In the full RGB image, the component occupies only a small part of the field of view and is surrounded by rails, jig regions, reflections, and background texture. When the image is resized for detection, small defect patterns can lose relative scale and become difficult to distinguish from these surrounding structures. The FSE and HGRG stages instead localize the component from the ToF intensity response and crop the corresponding region in the RGB image. As a result, a larger fraction of the detector input is occupied by the surface under inspection, which helps preserve local intensity and texture cues associated with defects.

This effect is particularly relevant to the Damage class, which includes scratches, dents, and local indentations. Such defects are often thin or irregular and can resemble machining marks or specular patterns on the metal surface. Cropping the image around the component retains these features at a larger relative scale before resizing, which is consistent with the observed improvement in Recall and mAP@50–95.

The ROI stage is implemented as preprocessing and does not alter the YOLO26 backbone, detection head, or loss function. It can therefore be placed upstream of an existing RGB detector. This additional localization step increases the average processing time from 13.2 to 45.9 ms/image, but it remains much faster than the sliding-window SAHI configuration used in the comparison. The selected FSE thresholds and ROI padding were also kept fixed across the dataset; a local sensitivity check showed stable ROI quality around these values, with degradation only when thresholding or padding became more aggressive.

Several limitations should be considered when interpreting the results. Although the dataset consists of actual manufactured components and manufacturing defects, all images were acquired in a controlled in-house setup. The reported performance therefore applies to the inspection configuration evaluated here and does not by itself establish generalization to other production lines. Broader validation will require external data collected with different materials, illumination conditions, sensor installations, and factory environments. The present experiments also assume one component per image and a fixed sensor geometry. When multiple components are close together or overlap, their ToF foreground regions may merge and connected-component analysis may no longer separate them reliably. In addition, larger component-height variations, sensor repositioning, or calibration drift can increase the error of the single homography used for ToF-to-RGB mapping.

Future work will therefore focus on instance separation for multi-component scenes and on local or multi-plane calibration for cases with larger 3D variation. Robustness to changes in illumination, sensor noise, and material properties should also be evaluated under less controlled production conditions.

## 6. Conclusions

This study proposed a cross-modal preprocessing framework that combines ToF intensity-based foreground support extraction and homography-guided object-adaptive ROI generation for metal surface defect detection. The proposed method uses ToF intensity dropout patterns as structural cues for component localization and projects the extracted support onto the RGB coordinate system to generate object-adaptive ROIs for defect detection. Experimental results showed that the proposed method improved mAP@50 from 0.585 to 0.796 and mAP@50–95 from 0.350 to 0.517 compared with the baseline YOLO26, while achieving Precision of 0.828 and Recall of 0.745. These results demonstrate that stabilizing the input ROI around the component before defect detection is effective for improving metal surface defect detection performance, even without modifying the detector architecture.

Future work will evaluate the proposed method in environments with multiple simultaneous components, diverse illumination and sensor noise conditions, and larger component height variations. The framework will also be extended toward a more generalized industrial inspection system.

## Figures and Tables

**Table 1:** Performance comparison for selecting the baseline defect detector.

Model	Precision	Recall	mAP@50	Processing Time (ms/Image)
Gold-YOLO [32]	0.500	0.88	0.479	81.3
YOLO11 [33]	0.675	0.583	0.572	13.2
YOLO26 [34]	0.704	0.588	0.585	13.2

**Table 2:** Performance comparison according to ROI generation strategy using the same YOLO26 detector.

Model	ROI Generation Strategy	Precision	Recall	mAP@50	Processing Time (ms/Image)
YOLO26 [34]	Full-size image	0.704	0.588	0.585	13.2
YOLO26 + SAHI [35]	Sliding window	0.282	0.323	0.479	3642.6
Proposed method	ToF-guided ROI	0.828	0.745	0.796	45.9

**Table 3:** Repeated-run stability comparison between the baseline YOLO26 and the proposed method.

Model	mAP@50					
	Run 1	Run 2	Run 3	Run 4	Run 5	Mean ± Std
YOLO26 [34]	0.598	0.560	0.580	0.592	0.597	0.585 ± 0.016
Proposed method	0.792	0.807	0.777	0.794	0.810	0.796 ± 0.013

**Table 4:** Quantitative comparison with existing object detection and preprocessing-based methods.

Model	Precision	Recall	mAP@50	mAP@50–95	Processing Time (ms/Image)
**YOLO26** [34]	0.704	0.588	0.585	0.350	13.2
**YOLO26 + SAHI** [35]	0.282	0.323	0.479	0.305	3642.6
**RT-DETR** [36]	0.635	0.583	0.575	0.356	39.7
**DEIM** [37]	0.767	0.198	0.476	0.260	56.2
**AIE and ESE** [38]	0.790	0.502	0.528	0.306	371.1
**Proposed method**	0.828	0.745	0.796	0.517	45.9

## Data Availability

The raw data supporting the conclusions of this article will be made available by the authors on request.
